# Soluble DLL1 as an Indicator of acute kidney injury and postoperative delirium following cardiac surgery: a secondary analysis of a prospective study

**DOI:** 10.1186/s13741-025-00570-4

**Published:** 2025-08-07

**Authors:** Thomas Simon Zajonz, Fabian Edinger, Melanie Markmann, Anna-Lena Schreiner, Frauke Beckert, Markus A. Weigand, Florian Uhle, Bernd Niemann, Michael Sander, Christian Koch, Emmanuel Schneck

**Affiliations:** 1 Department of Anesthesiology, Operative Intensive Care Medicine and Pain Therapy, University Hospital of Giessen and Marburg, Giessen, Germany; 2 Department of Anesthesiology, Heidelberg University Hospital, Heidelberg, Germany; 3 Department of Adult and Pediatric Cardiac and Vascular Surgery, University Hospital Giessen and Marburg Campus Giessen, Giessen, Hessen, Germany

**Keywords:** Biomarkers, Risk, Renal insufficiency, Inflammation, Postoperative cognitive complications, Neuropsychological tests, Delirium surgery

## Abstract

**Background:**

Acute kidney injury (AKI) displays a common complication after cardiac surgery and must be diagnosed as early as possible. Soluble delta-like protein 1 (sDLL1) was originally evaluated as a sepsis biomarker but might also indicate other adverse outcomes. This study aims to investigate sDLL1 levels, examining its potential relationship with AKI and postoperative delirium (POD) after cardiac surgery and its predictive value.

**Methods:**

This secondary analysis of a prospective observational trial included elective cardiac surgery patients. ELISA was used for the quantification of sDLL1. Statistical analysis involved repeated measures ANOVA and Pearson’s correlation to assess associations between sDLL1 levels, renal, and inflammatory parameters. Receiver operating curves were used for prediction analysis.

**Results:**

Ninety patients were included in the study. Compared to patients without AKI, those with AKI (6.1%) showed significantly elevated plasma levels of sDLL1 postoperatively (no AKI 6308.49 [5121.27–7955.28], AKI 7,714.77 [7151.06–10,514.73] ng/mL; *p* = 0.01). Postoperative sDLL1 levels showed only a low predictive value for AKI (AUCROC 0.63, sensitivity 0.91, specificity 0.53). Postoperative sDLL1 measurements were also significantly elevated in patients with POD (23.3%). Further, postoperative sDLL1 plasma levels showed a moderate prediction for the identification of POD (AUCROC 0.72, sensitivity 0.64 specificity 0.73).

**Conclusion:**

This study demonstrates that sDLL1 provides moderate predictive value for AKI and POD after cardiac surgery and may provide valuable insights into postoperative complications. sDLL1 levels increase independently of CPB type, suggesting a role in the inflammatory response to the cardiopulmonary bypass and surgical stress rather than specific renal injury.

**Trial registration:**

DRKS00010959.

**Supplementary Information:**

The online version contains supplementary material available at 10.1186/s13741-025-00570-4.

## Background

In 2019, soluble Delta-like protein 1 (sDLL1), a canonical notch ligand, was identified as a promising diagnostic biomarker due to its discriminatory potential, particularly in the context of sepsis (Gallenstein et al. 2023; Hildebrand et al. 2019; Schneck et al. 2022). sDLL1, part of the Delta/Jagged family of transmembrane proteins, is involved in the activation of intracellular pathways during embryonic angiogenesis and hematopoiesis (Bray 2016; Kovall et al. 2017). While sDLL1 has been predominantly studied in cancerous and infectious diseases (Capaccione and Pine 2013; Hildebrand et al. 2018; Penton et al. 2012), recent research has highlighted its role in sepsis (Gallenstein et al. 2023; Hildebrand et al. 2019; Hölle et al. 2023; Schneck et al. 2022). sDLL1 interacts with human monocytes, triggering a cellular immune response via notch signaling and releasing proinflammatory cytokines (Hildebrand et al. 2018). Additionally, it contributes to sepsis-induced endothelial damage, leading to impaired vascular barrier function (Joffre et al. 2020; Moll et al. 2021). The notch signaling pathway has garnered interest in sepsis research due to its influence on T-cell dysfunction and potential role in sepsis-induced immunosuppression (Jin et al. 2019; Pan et al. 2015; Venet and Monneret 2018). In septic patients, sDLL1 levels showed superior diagnostic accuracy compared to established markers such as CRP, leukocytes, and procalcitonin (PCT) (Hildebrand et al. 2019).

Own data suggested that sDLL1 might also be influenced by renal function (Schneck et al. 2022). This has been demonstrated in septic patients for the first time and must be confirmed by further studies. For this reason, we aimed for another study collective offering a high risk for renal dysfunction. Patients undergoing cardiac surgery are at an increased risk for suffering perioperative acute kidney injury (AKI) and were therefore of interest. The incidence of AKI is described as being up to 40% after cardiac surgery, causing significant impact on the patients’ long-term morbidity and mortality (Horne et al. 2023; Ortega-Loubon et al. 2022; Srivastava et al. 2012; Vandenberghe et al. 2015). It is of high importance that AKI is diagnosed as early as possible to provide adequate treatment (Yu et al. 2023).

In our previous study, we compared septic patients with patients either undergoing cardiac or major abdominal surgery (Schneck et al. 2022). sDLL1 increased already at the postoperative time point after cardiac surgery and increased further over the first 24 h, which was not seen in abdominal surgery. For this reason, questions were raised if it was connected to renal function (measured as creatinine, urea and glomerular filtration rate) and if it could be used as a biomarker for AKI. Therefore, this study aims to investigate sDLL1 levels in elective cardiac surgical patients to explore its potential relationship with renal function and its predictive value for the detection of AKI.

As a secondary aim, this study seeks to explore the potential association of sDLL1 with other adverse events following cardiac surgery. Given its established role as a sepsis biomarker, the first objective is to investigate the relationship between sDLL1 and postoperative infections.

Additionally, the study will examine its association with postoperative delirium (POD), since notch signaling, which is regulated by DLL1, plays a key role in neuronal development and the regulation of neuronal apoptosis induced by neuro-inflammation. This is of particular interest, as, in addition to surgical trauma, inflammation plays a pivotal role in the development of POD and POCD after cardiac surgery (Liu et al. 2018; Staicu et al. 2025). The inflammatory response can impair brain function by disrupting synaptic plasticity and promoting neuroinflammation (Sekine and Uchino 2017). Furthermore, this is characterized by elevated levels of proinflammatory cytokines and inflammatory biomarkers, which have been shown in clinical studies to correlate with the occurrence of POD and POCD (Sekine and Uchino 2017). While the underlying mechanisms are complex and multifactorial, targeting perioperative inflammation represents a promising approach to reduce the risk of POD and POCD. In a randomized controlled trial, Glumac et al. demonstrated that preoperative administration of dexamethasone significantly reduced both the incidence and severity of early POCD in patients undergoing cardiac surgery (Glumac et al. 2017). This benefit was accompanied by a marked reduction in systemic inflammatory response syndrome and lower postoperative C-reactive protein levels, indicating that the anti-inflammatory effects of corticosteroids likely contributed to the cognitive protection observed. Notably, the involvement of sDLL1 in postoperative delirium has not yet been investigated (Ables et al. 2011).

The primary aim of this study is to investigate whether perioperative sDLL1 kinetics reflect renal dysfunction and correlate with the development of AKI after cardiac surgery. Additionally, we explored whether elevated or dynamic changes in sDLL1 levels are associated with the occurrence of postoperative infections or POD.

## Methods

### Study design

This study presents a secondary analysis of an observational trial at a tertiary university hospital (Zajonz et al. 2023). It was registered in the German Clinical Trials Register (trial registration: DRKS00010959) and performed with permission of the local ethics committee (Justus Liebig University Giessen, Giessen, Germany; approval number AZ: 30/16). Written informed consent was obtained from all patients. The study was conducted according to the principles of the Declaration of Helsinki (2013). The methods and results are presented according to the Strengthening the Reporting of Observational Studies in Epidemiology (STROBE) guidelines (von Elm et al. 2008).

### Study endpoints

The primary study goal objective was to quantify the amount of sDLL1 during the perioperative period and to identify a correlation with renal damage defined as increase to or above stage I by the definition of KDIGO (Kellum and Lameire 2013). Further, kidney parameters are correlated with the concentration of sDLL1 as a surrogate of renal function.

Secondary outcome parameters included postoperative infections (defined as pneumoniae, catheter-related bloodstream infections [CRBSI], and urogenital infections) and the development of POD. Secondary outcome parameters included postoperative infections, defined as pneumonia, catheter-related bloodstream infections, and urogenital infections. Pneumonia was diagnosed based on the presence of new or progressive infiltrates on chest imaging, combined with at least two of the following clinical criteria: fever, leukocytosis or leukopenia, purulent respiratory secretions, and increased oxygen requirements. CRBSI was defined by systemic signs of infection in a patient with a central venous catheter, along with microbiological evidence of the same pathogen in blood. Urinary tract infections were diagnosed based on fever, in combination with laboratory findings, including significant bacteriuria, with or without positive urine cultures. After extubation, POD development was monitored for 7 days using the Intensive Care Delirium Screening Checklist (ICDSC) and Confusion Assessment Method for the Intensive Care Unit (CAM-ICU). A patient with a positive result with either instrument was considered to have POD.

#### Patient recruitment

Patients were recruited between September 2016 and January 2020. Patients were included if elective coronary artery bypass graft (CABG) surgery with postoperative intensive care treatment was performed. Further, inclusion criteria included age ≥ 18 years, elective on-pump CABG surgery, and the ability to communicate in German or English. Patients were excluded in case of missing consent, denial of participation, pregnancy, preoperative atrial fibrillation, severe bradycardia (< 60 bpm; types: sinus bradycardia, atrial fibrillation with low frequency, nodal rhythm, and second- or third-degree atrioventricular block), acute infection before surgery, pre-existing autoimmune disease, immunomodulatory medication, left ventricular ejection fraction < 30%, and renal insufficiency (Kidney Disease Improving Global Outcome score > 2). Further exclusion criteria were pseudocholinesterase deficiency, cognitive dysfunction (e.g., history of schizophrenia, other severe psychiatric conditions, or dementia with inability to adequately answer the CAM-ICU or ICDSC), and recent or persistent neurological impairment (e.g., acute cerebral infarction, intracranial bleeding, or acute meningitis in the last 3 months prior to study inclusion leading to inability to answer the CAM-ICU or ICDSC).

### Management of cardiopulmonary bypass

The patients in this non-interventional study underwent standard anesthetic induction with sufentanil (0.25–0.5 μg/kg), etomidate (0.1–0.2 mg/kg), and pancuronium (0.05–0.1 mg/kg). Anesthesia was maintained using propofol (3 mg/kg/h) and sufentanil (0.3–1 μg/kg/h). Central venous access was established via the internal jugular vein, and arterial blood pressure was monitored through the radial artery.

Both conventional CPB (cCPB) and minimized extracorporeal circulation (MiECC) utilized the S5 Heart–Lung Machine (LivaNova, London, UK) equipped with membrane oxygenators. MiECC employed the CAPIOX® FX 15 Oxygenator (Terumo, Tokyo, Japan), while cCPB used the Inspire® 6 F Oxygenator (LivaNova, London, UK) alongside a specialized perfusion tubing system (LivaNova, London, UK). For cCPB, a roller pump and an additional reservoir (LivaNova, London, UK) were implemented, whereas MiECC operated with a centrifugal pump, without the need for a reservoir or hemofilter.

The priming solution for the circuits consisted of 1 L of crystalloid (Sterofundin Iso®, Braun, Melsungen, Germany), 250 mL of 15% mannitol (Seraq-Wiessner, Naila, Germany), and 50 mL of 20% albumin (CSL Behring, Marburg, Germany). For MiECC, 10,000 I.U. of heparin was added, while only 2500 I.U. was required for cCPB.

### Sample processing

Blood was collected at six time points: immediately after induction of anesthesia (T1); 15 (T2) and 60 (T3) min after the commencement of CPB, respectively; and 15 (T4) and 120 (T5) min after the end of CPB after admission to the intensive care unit (ICU). Last, blood was drawn through the arterial line with the routine laboratory control at the early morning of the first postoperative day (T6). Blood was collected in ethylenediaminetetraacetic acid (EDTA) tubes (approximately 20 mL). Plasma samples were stored at – 80 °C. Clinical and laboratory data were obtained from the patient data management system (IMESO GmbH, Giessen, Germany).

### Quantification of sDLL1

The plasma concentration of sDLL1 was quantified using a commercially available enzyme-linked immunosorbent assay (ELISA) kit (RayBiotech Life, Inc., Norcross, USA) according to the manufacturer’s instructions. To fit measurements into the calibration curves and simultaneously reduce interfering matrix effects, all samples were diluted 1:30 (or higher if demanded by the concentration) with the supplied Assay Diluent A prior to the measurements. An ELx808 microplate reader (BioTek Instruments, Inc., Winooski, USA) was used for absorbance measurements, with a subsequent automatized calculation of concentrations by the corresponding Gen5 software (BioTek Instruments, Inc., Winooski, USA).

### Statistical analysis

All numeric data were expressed as median and interquartile range (IQR). The analysis of variations in plasma sDLL1 levels across different time points was performed by repeated measures ANOVA, followed by the Tukey HSD test. The comparison of postoperative sDLL1 levels for patients with POD or AKI versus those without, respectively, was done by Wilcox Test. Potential correlation between plasma sDLL1 levels and inflammatory and renal parameters was analyzed by Pearson’s correlation coefficient. Influence of sDLL1 on POD or AKI has been examined by logistic regression analysis while receiver operating characteristic (ROC) curve analyses were used for the calculation of area under the ROC curve (AUCROC), sensitivity, and specificity of inflammatory and renal parameters. *p*-values < 0.05 were considered statistically significant and set as two-sided. All data were stored in an external database (Microsoft Excel, Redmond, USA). Data were analyzed using R version 4.3.2 (2023–10-31; www.r-project.org).

## Results

### Study cohort

Of the 100 patients included in the primary study, sufficient sample volume for this secondary analysis was achievable for 90 patients. The characteristics of these 90 patients are shown in Table 1. The only difference between the study groups was the incidence of myocardial infarction within the 90 days prior to surgery, with a higher occurrence in the cCPB group (*p* < 0.001). No correlation was found between underlying diseases and sDLL1 concentrations, except for diabetes (logistic regression, *p* < 0.001). No patient died during the observational period. Plasma levels of sDLL1 were correlated with the duration of anesthesia (correlation coefficient = 0.30 [0.08–0.49]; *p* = 0.007).

**Table 1:** Baseline characteristics

*Parameters*	*All patients*	*MiECC*	*cCPB*
Age (years)	64 [57.25–71]	61.5 [56.75–71]	67.5 [59–72.5]
Male sex, *n* (%)	79 (87.8)	44 (91.7)	35 (83.3)
BMI (kg/m^2^)	29.04 [26.02–32.13]	29.2 [27.74–31.85]	28.41 [24.97–32.98]
EuroSCORE	1.06 [0.76–1.32]	0.96 [0.76–1.25]	1.13 [0.77–1.52]
***Pre-existing diseases***			
Angina pectoris, *n* (%)	59 (67.0)	34 (72.3)	25 (61.0)
Arterial hypertension, *n* (%)	78 (87.6)	43 (89.6))	35 (85.4)
Acute myocardial infarction, *n* (%)	22 (24.7)	9 (18.8)	13 (31.7)
Myocardial infarction within the last 90 days prior to surgery, *n* (%)	19 (21.6)	4 (8.5)	15 (36.6)
Concurrent valvular disease			
Stroke, *n* (%)	11 (12.4)	2 (4.2)	9 (22.0)
Diabetes, *n* (%)	35 (39.3)	18 (37.6%)	17 (41.5%)
Chronic obstructive pulmonary disease (> 1), *n* (%)	10 (11.2)	5 (10.4)	5 (12.2)
Smoker, *n* (%)	54 (60.7)	28 (58.3)	26 (63.4)
Alcohol abuse, *n* (%)	2 (2.2)	1 (2.1)	1 (2.4)
**Process times**			
Duration of anesthesia (min)	201.5 [172.56–233.5]	192.75 [168.19–224]	214.88 [189–248.5]
Duration of CPB (min)	83 [72–105]	78 [68.75–92.25]	90 [77–112.5]
Duration of invasive ventilation (min)	809 [605.25–1079.5]	780.5 [576–1077.75]	834 [655–1144.75]

*Abbreviation: CPB *Cardiopulmonary bypass

### Time courses of sDLL1

**Fig. 1:**
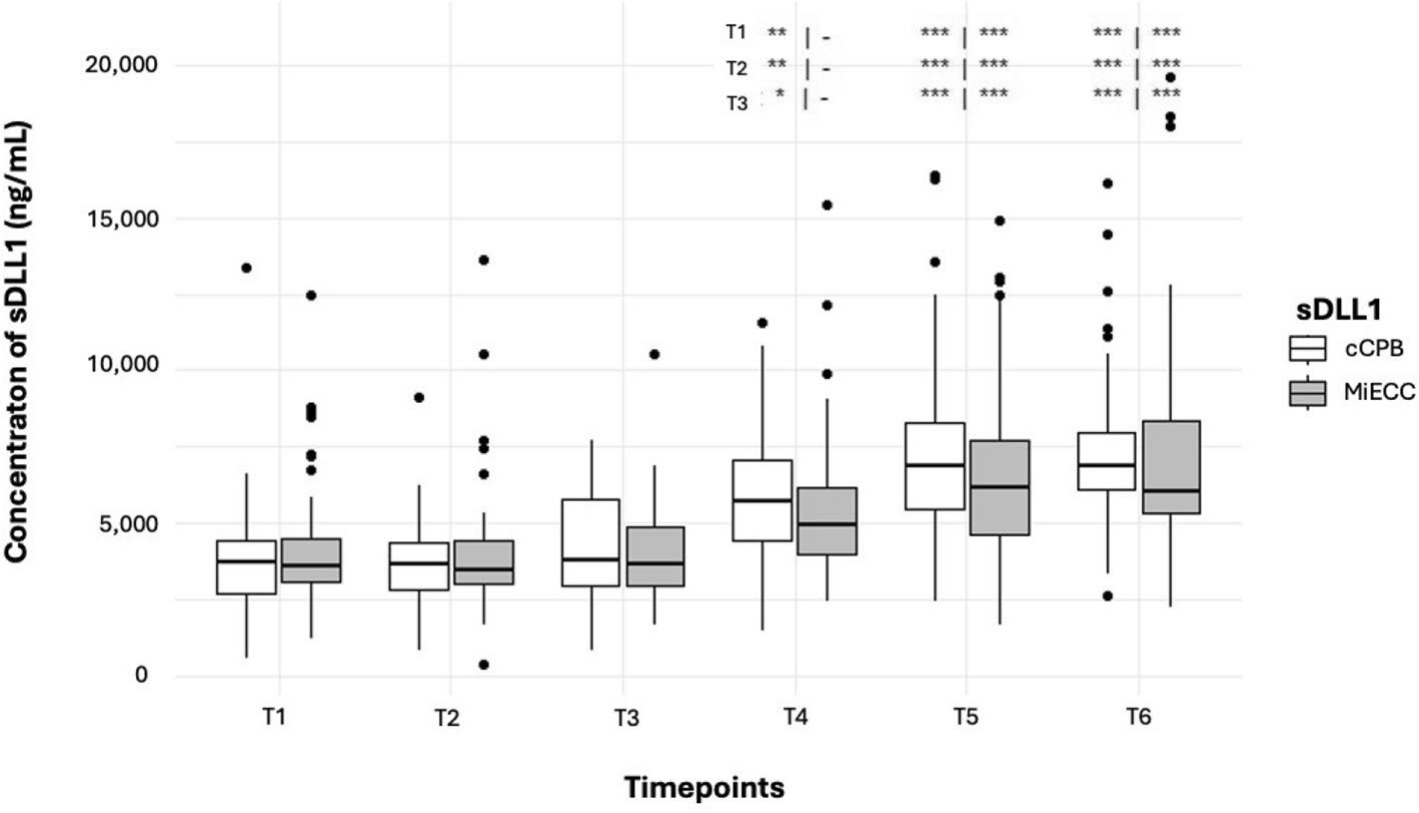
Boxplots showing the time course of sDLL1 in dependence on MiECC and cCPB. Asterisks refer to the boxplots below and denote significant differences compared to T1, T2, and T3 (cCPB | MiECC; ***p < 0.001, **p < 0.01 *p < 0.05). *Abbreviations*: cCPB = conventional cardiopulmonary bypass, MiECC = minimized extracorporeal circulation, sDLL1 = soluble Delta-like canonical Notch ligand

Independently of the type of CPB, the amount of sDLL1 increased significantly in comparison to the preoperative values. The increase began 15 min after the ending of the CPB (T4), independently of the use of MiECC or cCPB (Fig. 1, Table 2). Summarized data of the postoperative time points (T5 & T6, MiECC and cCPB) showed significant elevation of sDLL1 compared to T1–T4 (all *p* < 0.001), whereas sDLL1 is already elevated close to the end of CPB, respectively (T4 compared to T5 and T6: *p* = 0.006 and *p* < 0.001).

**Table 2:** Overview on the results of the sDLL1 quantification

	*sDLL1 (ng/mL)* *All patients*	*sDLL1 (ng/mL)* *POD*	*sDLL1 (ng/mL)* *non-POD*	*p*-*value*
*T1 (preoperative)*	3647.82 [2898.75–4435.47]	3825.60 [2680.14–5245.14]	3631.51 [3034.72–4287.95]	1.00
*T2 (15´ CPB)]*	3472.65 [2899.05–4416.65]	3780.93 [3029.38–5494.32]	3472.65 [2880.16–4181.87]	1.00
*T3 (60´ CPB)*	3820.77 [2945.75–5297.3]	4785.54 [3815.94–6181.35]	3615.21 [2847.3–4773.81]	0.98
*T4 (15´ after end of CPB)*	5248.54 [4074.91–6982.27]	5970.69 [4677.93–8163.12]	5074.02 [4027.88–6589.26]	0.98
*T5 (120´ after end of CPB)*	6370.77 [5043.51–8106.96]	7896.15 [6557.85–11,868.75]	6027.78 [4767.7–7330.26]	0.04
*T6 (1st day postoperative)*	6511.14 [5441.26–8218.6]	8282.25 [6468.03–11364.72]	6106.2 [5177.92–7514.33]	< 0.001
*All postoperative time points *	6490.47	6143.50	6903.57	n.s.
*summarized (T5-T6)*	[5233.52-8224.22]	[5063.32-8185.73]	[5545.01-8178.04]	

Measurements are given as median [interquartile range]; *p*-value is given for comparison POD vs. non-POD

*Abbreviations*: *POD* = postoperative delirium (three patients with preoperative delirium included), *sDLL1 *soluble Delta-like canonical Notch ligand 1

Overall, sDLL1 concentrations were not significantly lower in patients undergoing MiECC compared to cCPB (*p* = 0.75). However, a significant increase in sDLL1 levels was observed with cCPB as early as T4 (cCPB T4 vs. T1: *p* < 0.01, T2: *p* < 0.01, and T3: *p* < 0.05; T5 and T6 vs. T1, T2, and T3: *p* < 0.001), while with MiECC, a significant increase was only observed at T5 and T6 (MiECC T5 and T6 vs. T1, T2, and T3: *p* < 0.001). Absolute values are shown in Table 1.

### Primary endpoint analysis

#### sDLL1 as potential predictive biomarker for AKI

**Fig. 2:**
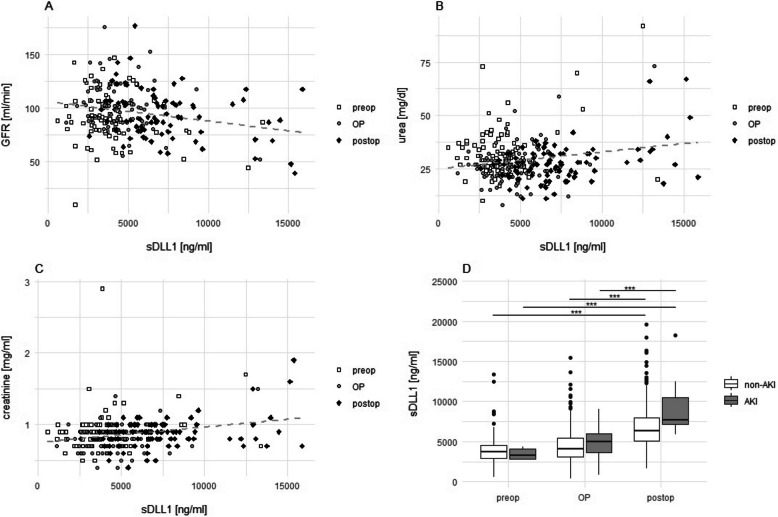
Scatterplots showing the correlation of sDLL1 with the GFR (**A**), the concentration of urea (**B**) and creatinine (**C**).** D** demonstrates boxplot diagrams of sDLL1 at different time frames in dependence on the occurrence of an AKI. Abbreviations: GFR = glomerular filtration rate; OP = intraoperative (T2-4); preop = preoperative (T1); postop = postoperative (T5 and T6); sDLL1 = soluble Delta-like canonical Notch ligand

Altogether 28 patients (31.1%) suffered from an AKI stage 1 or higher (AKI 1 *n* = 24 [26.7%]; AKI 2 *n* = 3 [3.3%]; AKI 3 *n* = 1 [1.1%]; Fig. 2D). Postoperative sDLL1 levels in patients with AKI were statistically significantly higher than those in patients without kidney damage (no AKI: 6150.99 [5115.12–7540.8], AKI 7515.54 [5852.94–10,890.24]; *p* = 0.002). Urea decreased significantly on day 1 after surgery compared to the baseline value; however, without a clinical relevance (baseline urea [T1] = 30 [26–37], postoperative [T5 and T6] = 27 [22–32]; *p* = 0.004). The averaged pre-, intra-, and postoperative plasma concentration of sDLL1 showed a correlation with the renal parameters creatinine, urea, and GFR, especially in the postoperative period (all timepoints: creatinine: correlation coefficient = 0.25, adj. *r*^2^ = 0.06, *p* < 0.001; urea: correlation coefficient = 0.20, adj. *r*^2^ = 0.04, *p* < 0.001; glomerular filtration rate [GFR]: correlation coefficient = − 0.21, *r*^2^ = 0.05, *p* < 0.001; post-operative timepoints [T5 and T6]: creatinine: correlation coefficient = 0.39, adj. *r*^2^ = 0.15, *p* < 0.001; urea: correlation coefficient = 0.41, adj. *r*^2^ = 016, *p* < 0.001; GFR: correlation coefficient = − 0.31, *r*^2^ = 0.09, *p* < 0.01; Fig. 2A–C). However, postoperative sDLL1 levels provided only limited value in predicting AKI (Table 3).

**Table 3:** Results of the logistic regression and ROC analysis for the prediction of AKI of the postoperative timepoints (T5 and T6)

Parameter	AUCROC [95% CI]	Cutoff value	Specificity	Sensitivity	*p-*value
**sDLL1 (ng/mL)**	0.63 [0.654–0.73]	7587.42	0.76	0.49	0.003
**Creatinine (mg/dL)**	0.72 [0.59–0.84]	0.95	0.84	0.58	0.006
**Glomerular filtration rate (mL/min)**	0.76 [0.63–0.89]	85.5	0.81	0.67	< 0.001
**Urea (mg/dL)**	0.66 [0.53–0.80]	28.5	0.72	0.61	0.035

ROC analysis included all postoperative time points of the surgical patients

*Abbreviations: AKI *Acute kidney injury*, AUCROC *Area under the ROC curve*, 95% CI *95% confidence interval*, ROC *Receiver operating characteristic*, sDLL1 *soluble Delta-like canonical Notch ligand

### Secondary endpoint analysis

#### sDLL1 as potential predictive biomarker for infection

No patient developed a sepsis, catheter-related bloodstream infection, or urogenital infection. Pneumonia was the most common infectious disease with three (3.3%) cases. In these patients, postoperative sDLL1 plasma levels were not significantly increased compared to patients without pneumonia (without pneumonia 6419.4 [5126.95–8256.04]; with pneumonia: 7235.43 [7010.65–7457.26]; *p* = 0.36). Data of sDLL1 and the other inflammatory parameters are shown in Supplementary Table S1.

### sDLL1 as a potential biomarker for postoperative delirium

**Fig. 3:**
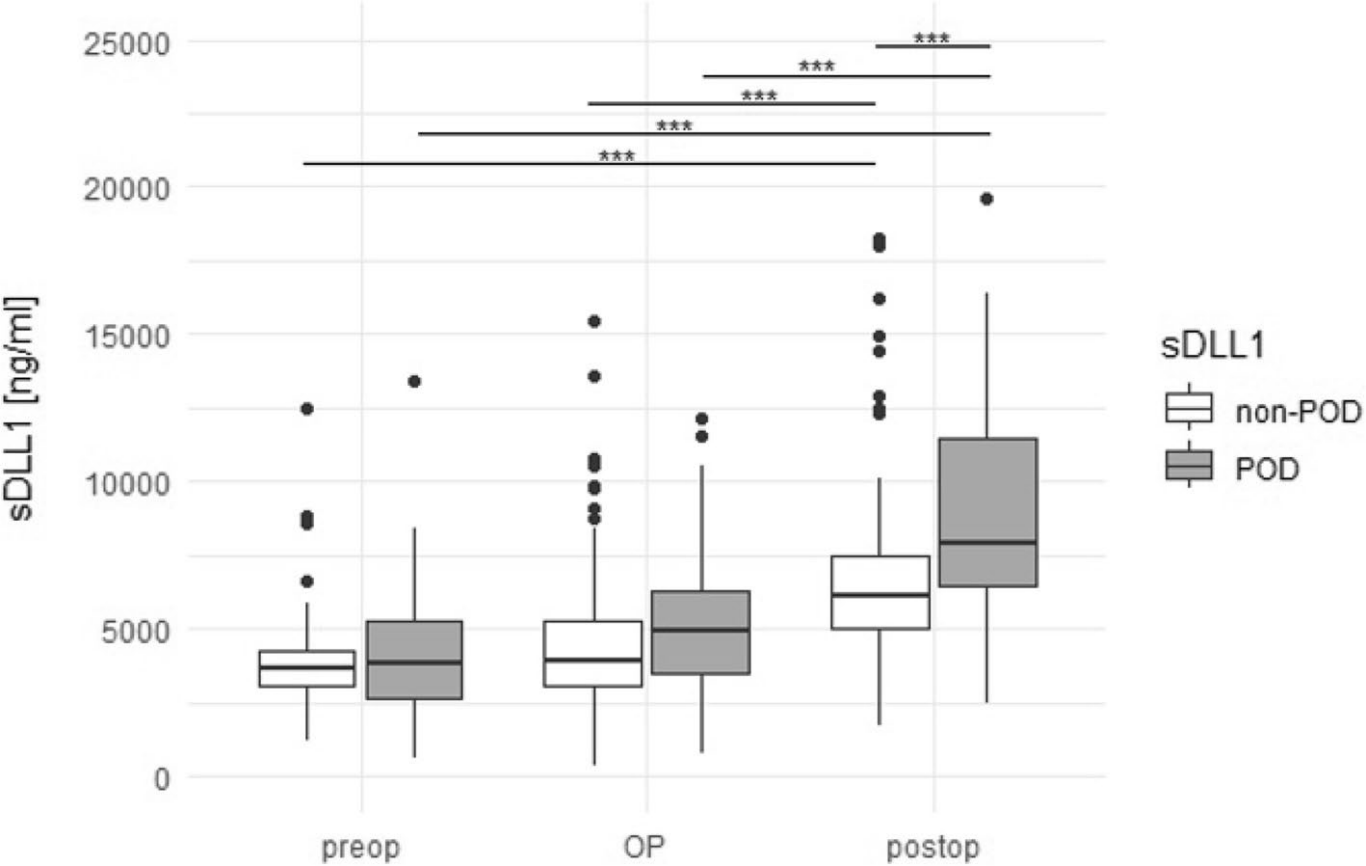
Boxplot demonstrating differences in the sDLL1 plasma levels in dependence on the occurrence of POD. Abbreviations: OP = intraoperative (T2-4); preop = preoperative (T1); postop = postoperative (T5 and T6); POD = postoperative deliriu

**Fig. 4:**
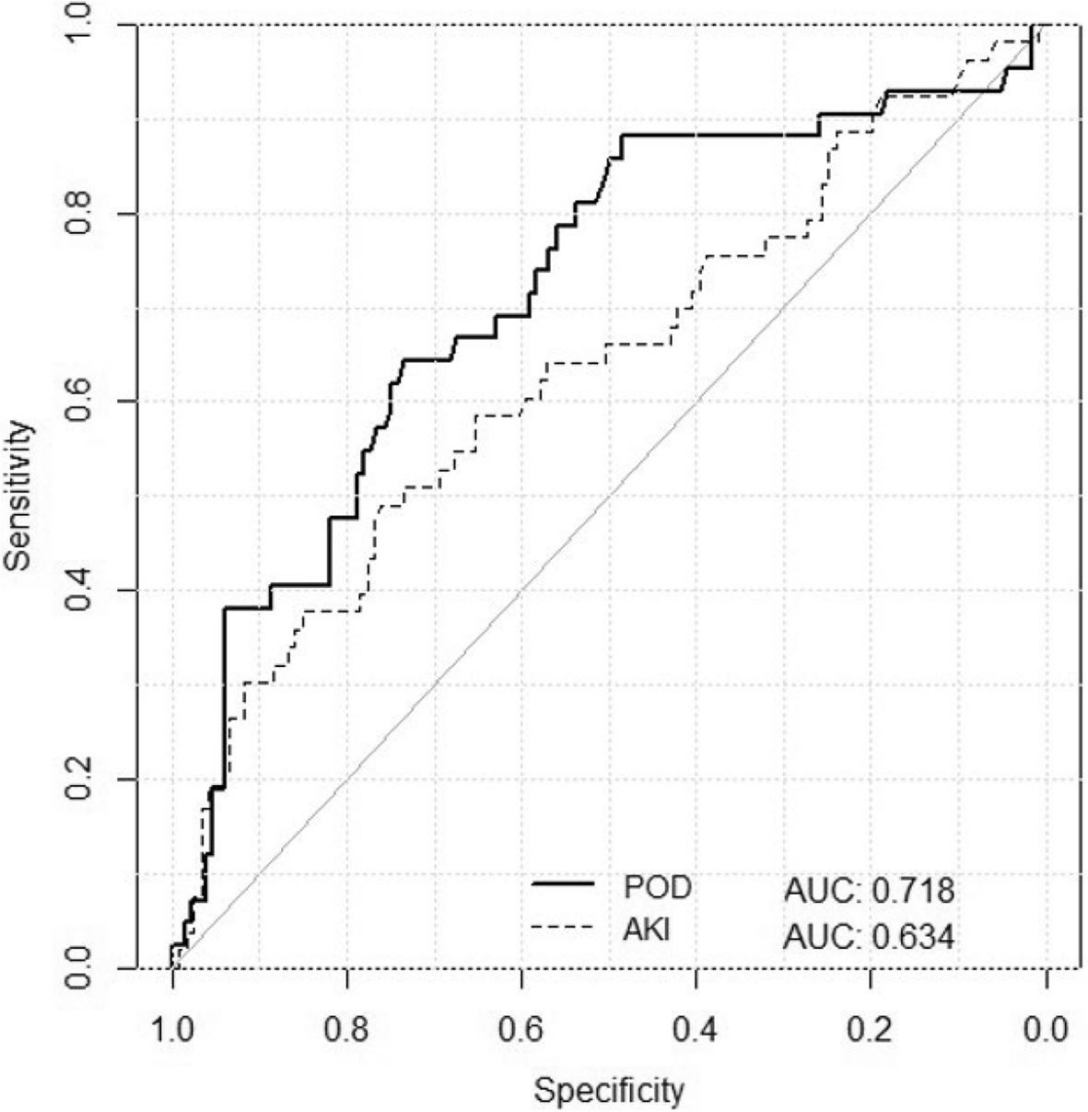
ROC curve demonstrating the predictive power of postoperative sDLL1 plasma levels for positive diagnosis of delirium (POD) and kidney damage (AKI). Abbreviations: AKI = acute kidney injury; AUC = area under the curve; POD = postoperative delirium

Overall, 21 (23.3%) patients developed POD, while three of those had delirium already prior to surgery. Postoperative (T5 and T6) sDLL1 plasma levels were significantly elevated in patients with POD (Table 4, Fig. 3) and showed a moderate prediction for the identification of POD (AUCROC 0.72, sensitivity 0.64 specificity 0.73, *p* < 0.001, Fig. 4). A trend towards predictiveness can already be seen during surgery (*p* < 0.05).

**Table 4:** Overview on the results of the sDLL1 quantification in patients with and without POD

	*sDLL1 (ng/mL)* *All patients*	*sDLL1 (ng/mL)* *POD*	*sDLL1 (ng/mL)* *non-POD*	*p*-*value*
*T1 (preoperative)*	3647.82 [2898.75–4435.47]	3825.60 [2680.14–5245.14]	3631.51 [3034.72–4287.95]	1.00
*T2 (15´ CPB)]*	3472.65 [2899.05–4416.65]	3780.93 [3029.38–5494.32]	3472.65 [2880.16–4181.87]	1.00
*T3 (60´ CPB)*	3820.77 [2945.75–5297.3]	4785.54 [3815.94–6181.35]	3615.21 [2847.3–4773.81]	0.98
*T4 (15´ after end of CPB)*	5248.54 [4074.91–6982.27]	5970.69 [4677.93–8163.12]	5074.02 [4027.88–6589.26]	0.98
*T5 (120´ after end of CPB)*	6370.77 [5043.51–8106.96]	7896.15 [6557.85–11,868.75]	6027.78 [4767.7–7330.26]	0.04
*T6 (1st day postoperative)*	6511.14 [5441.26–8218.6]	8282.25 [6468.03–11364.72]	6106.2 [5177.92–7514.33]	< 0.001

Measurements are given as median [interquartile range]; *p*-value is given for comparison POD vs. non-POD

*Abbreviations*: *POD *postoperative delirium (three patients with preoperative delirium included), *sDLL1 *soluble Delta-like canonical Notch ligand 1

## Discussion

This study revealed three main findings. First, it demonstrated the intra- and early postoperative dynamics of sDLL1, which have not yet been described. sDLL1 levels rose as early as 15 min after the end of CPB, regardless of whether MiECC or cCPB was used, indicating that the type of CPB did not influence sDLL1 release. Second, plasma concentrations of sDLL1 increased in the early postoperative phase, correlated with renal parameters, and provided moderate predictive value for AKI. Lastly, sDLL1 concentrations were elevated in patients who developed POD and showed also moderate prediction for identifying POD.

Since sDLL1 has been evaluated as a biomarker for gram-negative infections and sepsis, its behavior during cardiac surgery has hardly been investigated. This study suggests that sDLL1 is robust against preoperative confounders such as pre-existing diseases or medications. Only pre-existing diabetes correlated with increased sDLL1 concentrations, a finding not previously reported. Furthermore, neither the induction of anesthesia nor the initiation of CPB raised sDLL1 plasma levels immediately. Interestingly, it was not the duration of CPB but the duration of anesthesia that was associated with elevated sDLL1 levels, raising the question of whether CPB alone is responsible for sDLL1 release. A potential link between anesthesia and adverse outcomes could be the drug interactions or the occurrence of intraoperative hypotension, for example (Sun et al. 2018; Wesselink et al. 2018; Zarour et al. 2024). The finding that MiECC did not reduce circulating sDLL1 compared to cCPB suggests that CPB is not the sole contributor to sDLL1 release. This result was somewhat unexpected, as MiECC is known to reduce other inflammatory biomarkers (Liebold and Albrecht 2019; Remadi et al. 2006; Zajonz et al. 2022). Although our previous study did not describe the intraoperative course of sDLL1, it did measure levels post-cardiac surgery (Schneck et al. 2022). Compared to the current results, it is notable that overall sDLL1 concentrations were lower in the previous study, although the relative changes (doubling from preoperative to 24 h postoperatively) remained comparable. The cause of this discrepancy is unclear, as both studies included patients undergoing elective CABG surgery, with MiECC accounting for the lower sDLL1 concentrations.

The use of CPB in cardiac surgery has a profound impact on inflammatory processes of the human body. Exposure to artificial surfaces, ischemia–reperfusion injury, and the sheer physical stress induced by CPB collectively trigger a systemic inflammatory response. This heightened inflammatory state can significantly alter the levels of numerous biomarkers, often resulting in nonspecific elevations that obscure their diagnostic or prognostic value (Squiccimarro et al. 2022). The systemic inflammatory response triggered by CPB, including the activation of cytokines, complement pathways, and leukocytes, may elevate biomarker levels nonspecifically. The role of sDLL1 in the context of CPB is not yet well understood. However, since the Notch signaling pathway plays a key role in the development of cardiac and vascular tissue, it can be assumed that it is involved in the CPB-related inflammatory response (MacGrogan et al. 2018). One possible link is the interaction between metalloproteases and the Notch signaling family, including sDLL1. For instance, strong inflammation and cardiovascular disease lead to an upregulation of ADAM metalloproteases (A Disintegrin and Metalloproteinase, ADAM10/17), which cleave membrane-bound DLL1 (Dyczynska et al. 2007; Quillard and Charreau 2013). This process is further amplified by complement activation and damage-associated molecular patterns (DAMPs), which stimulate Toll-like receptors and activate NF-κB–dependent pathways, thereby enhancing both DLL1 expression and its proteolytic processing. Inflammatory cytokines such as tumor necrosis factor (TNF), interleukin-1β (IL-1β), and interferon-γ (IFN-γ) induce a shift in the expression pattern of Notch molecules in endothelial cells, which are also directly affected by CPB. This mechanism may contribute to post-CPB vascular leakage and other complications such as AKI.

The generalized inflammatory response can also reduce the specificity of AKI biomarkers, making it challenging to distinguish true renal injury from the inflammatory background noise caused by CPB. Consequently, the reliability of biomarkers as indicators of specific pathophysiological conditions is compromised, as their levels may reflect not only the underlying disease process but also the generalized inflammatory response induced by CPB. Consequently, markers such as neutrophil gelatinase-associated lipocalin (NGAL) and kidney injury molecule-1 (KIM-1), while potentially useful in detecting AKI, may reflect, particularly in less severe cases of AKI, the overall inflammatory status rather than isolated renal damage (Doi et al. 2013; Ho et al. 2015).

For this reason, the search for biomarkers for AKI is continuing. It was known that sDLL1 is robust to the influence of confounding factors such as pre-existing diseases and medications, which has also been demonstrated in this study. However, a previous own study showed a potential association of sDLL1 and disturbances of the renal function (Schneck et al. 2022). Therefore, the primary aim of this study was to investigate if sDLL1 was associated with the renal parameters and AKI. 31.1% of patients developed stage I AKI, and we demonstrated that sDLL1 correlated with renal parameters and was significantly elevated in patients with AKI compared to those without. Nevertheless, postoperative sDLL1 plasma levels demonstrated little predictive power for detecting AKI, with sensitivity and specificity comparable to that of GFR, creatinine and urea (sDLL: sensitivity 0.77, specificity 0.49). This result indicates that sDLL1 release might be triggered by the nonspecific inflammatory response induced by anesthesia, surgery, or CPB rather than a specific renal source. However, only the postoperative increase in sDLL1 correlated with renal damage, limiting its utility as a tool for preoperative risk assessment. Interestingly, the only prior study investigating the relationship between sDLL1 and AKI reported an AUCROC of 0.9 [0.82–0.9] for AKI detection in septic and postsurgical patients, suggesting synergistic effects between infectious disease and AKI (Schneck et al. 2022). It is possible that sepsis, which leads to much higher sDLL1 concentrations and more severe AKI, enhanced the sensitivity and specificity of sDLL1 for AKI detection. In summary, sDLL1 is most likely not suitable as a standalone biomarker for AKI; however, its association with renal function should be considered when using it as an infectious biomarker. Furthermore, it may have utility in multi-panel approaches that are also being explored for AKI detection following cardiac surgery (Kalisnik et al. 2022; Squiccimarro et al. 2022). It would be ideal to identify preoperative parameters, as this would allow for risk assessment before surgery rather than relying solely on intraoperative findings without the possibility of therapeutic actions (e.g., preconditioning).

In an exploratory approach, we set a secondary aim for the study to investigate the predictive capacity of sDLL1 for identifying POD. Surprisingly, our findings showed that postoperative sDLL1 concentrations were increased in patients with POD, and furthermore, that sDLL1 was predictive for its identification. This is the first study to describe this finding; therefore, it should be interpreted with caution until confirmed in subsequent studies. These findings may be explained by several factors. First, sDLL1 could serve as a biomarker for inflammatory processes associated with surgical stress. It is well established that surgical procedures trigger a systemic inflammatory response, which may stimulate the release of sDLL1. Given that POD is often linked to inflammatory and neuroinflammatory reactions, the elevated sDLL1 levels might indicate underlying pathophysiological processes (Jin et al. 2020). Additionally, sDLL1 may play a role in neuronal signaling and the regulation of cell adhesion (Ables et al. 2011; Gallenstein et al. 2023). Dysregulation of these processes could contribute to the development of POD by exacerbating neuroinflammatory mechanisms or impairing neuronal homeostasis. Lastly, even though sDLL1 seemed to be robust against confounding factors, it cannot be ruled out by one study that the association between increased sDLL1 and POD might also be influenced by patient characteristics such as age, cognitive impairments, or preoperative comorbidities, which could affect both sDLL1 levels and the risk of developing POD. These factors warrant further investigation in future studies to validate the role of sDLL1 as a biomarker for POD and to better understand the underlying mechanisms.

This study has several limitations. First, the relatively small sample size of the study limited its statistical power, particularly with regard to subgroup analyses such as infectious complications. Nonetheless, the findings provide valuable insights that may guide sample size estimations for future research, consistent with the exploratory nature of the study. The results highlight the relevance of further investigating sDLL1 dynamics during CPB and emphasize the need for larger, prospectively designed studies with a higher number of patients. Second, as a secondary analysis of an observational study, plasma concentrations of PCT were not available for all patients. However, since PCT kinetics typically develop after 24 h, a timepoint beyond the observational period, this limitation is of only minor relevance to the study (Aouifi et al. 1999). Furthermore, the observed increase in PCT levels was only modest. Additionally, due to the study’s design, it was not possible to measure sDLL1 concentrations in urine, which would have been of considerable interest. Third, the observation period was relatively short because of the study’s exploratory nature. Fourth, the inclusion period was prolonged due to competing studies; however, anesthetic approaches remained unchanged during this time frame. Finally, as previously noted, this study does not elucidate the underlying mechanisms of sDLL1 release in surgical intensive care patients.

## Conclusion

In conclusion, this study demonstrates that sDLL1 is associated with renal damage and POD following cardiac surgery and may support the prediction of POD. sDLL1 levels increase independently of CPB type, suggesting a primary role in the inflammatory response to surgical stress rather than indicating specific renal injury. These findings highlight the potential utility of sDLL1 for detecting AKI and POD; though further research is essential to confirm its relevance and clarify the mechanisms underlying its release in cardiac surgery patients. Particularly, the low predictive ability for detecting AKI warrants further investigation.

## Supplementary Information

Supplementary Material. Supplemental Table 1. Overview of the inflammatory parameters at different time points. Values are given as median [IQR]. Abbreviations: CRP = C-reactive protein; IL-6 = interleukin 6; IQR = interquartile range; N.A. = Not available; PCT = Procalcitonin.

## Data Availability

The datasets used and/or analyzed during the current study are available from the corresponding author on reasonable request.

## Abbreviations

AKI: Acute kidney injury
AUC ROC: Area under the curve of the receiver operator characteristic
CABG: Coronary artery bypass graft
CAM-ICU: Confusion Assessment Method for the Intensive Care Unit
cCPB: Conventional cardiopulmonary bypass
CPB: Cardiopulmonary bypass
CRBSI: Catheter-related bloodstream infections
EDTA: Ethylenediaminetetraacetic acid
ELISA: Enzyme-linked immunosorbent assay
ICDSC: Intensive Care Delirium Screening Checklist
IQR: Interquartile range
KDIGO: Kidney Disease Improving Global Outcomes
KIM-1: Kidney injury molecule-1
MiECC: Minimized extracorporeal circulation
NGAL: Neutrophil gelatinase-associated lipocalin
POD: Postoperative delirium
PCT: Procalcitonin
ROC: Receiver operating characteristic
sDLL1: Soluble Delta-like protein 1
STROBE: Strengthening the Reporting of Observational Studies in Epidemiology
