# Supplementary material for: Soluble DLL1 as an Indicator of acute kidney injury and postoperative delirium following cardiac surgery: a secondary analysis of a prospective study
Source: Perioper Med (Lond). 2025 Aug 7;14:85. doi: 10.1186/s13741-025-00570-4 (PMC12330148; doi:10.1186/s13741-025-00570-4)
Supplement: Supplementary file 1 — Supplementary Material. Supplemental Table 1. Overview of the inflammatory parameters at different time points. Values are given as median [IQR]. Abbreviations: CRP = C-reactive protein; IL-6 = interleukin 6; IQR = interquartile range; N.A. = Not available; PCT = Procalcitonin. [file 13741_2025_570_MOESM1_ESM.docx]

**Supplementary Material**

|  | **Leukocytes (number/µL)** | | | **CRP (mg/mL)** | | | **PCT (µg/L)** | | |
| --- | --- | --- | --- | --- | --- | --- | --- | --- | --- |
|  | **All**  **patients** | **MiECC** | **cCPB** | **All**  **patients** | **MiECC** | **cCPB** | **All patients** | **MiECC** | **cCPB** |
| **Pre-operative (T0)** | 7.55  [6.6 - 8.97] | 7.4  [6.3-8.9] | 7.7  [6.93-8.97] | 4  [1.15 - 11.5] | 3.17  [1.12-10.07] | 5.96  [1.32-13.3] | 0.58  [0.58-0.58] | 0.58  [0.58-0.58] | n.a. |
| **Operative (T1-3)** | 11.95  [8.07 - 15.05] | 12.3  [9.35-15.72] | 11.05  [7.4-14.17] | 2.78  [0.3 - 8.14] | 2.16  [0.25-8.36] | 3.21  [0.77-7.29] | n.a. | n.a. | n.a. |
| **Post-operative**  **(T4-6)** | 12.15  [9.9 - 14.12] | 12  [10.28-13.38] | 12.15  [9.43-14.43] | 91.12  [69.17 - 116.67] | 96.5  [71.2-115.91] | 87.31  [70.04-130.07] | 1.6  [0.6-2.1] | 1.85  [1.73-1.98] | 0.6  [0.55-2.2] |

**Supplemental Table 1** Overview of the inflammatory parameters at different time points. Values are given as median [IQR]. *Abbreviations: CRP = C-reactive protein; IL-6 = interleukin 6; IQR = interquartile range; N.A. = Not available; PCT = Procalcitonin*
